# Language does arithmetic: linguistic differences in children’s place-value processing

**DOI:** 10.1007/s00426-022-01653-3

**Published:** 2022-02-22

**Authors:** Christina Kraut, Silvia Pixner

**Affiliations:** Department of Psychology and Medical Sciences, UMIT Tirol-The Tyrolean Private University, Eduard-Wallnoefer-Zentrum 1, 6060 Hall in Tyrol, Austria

## Abstract

The representation and retrieval of multiplication facts is dependent on linguistic specificities such as number word inversion (i.e., 23 is spoken dreiundzwanzig in German which translates to three and twenty). Previous research has evaluated these language influences in adults. Now this study aims to follow-up on earlier findings and takes a closer look at inversion-related effects on place-value processing during multiplication in children. In a task of choice 46 children, either German- or Italian-speaking, had to pick the right answer out of two options for a given multiplication problem. Already established effects in adult participants such as decade-consistency and table-relatedness were also present in elementary school children, but different between the language groups. For decade-consistent items the effect of table-relatedness was larger for Italian-speaking students than for German-speaking. This indicates that the inversion property in the German language leads to those children putting less emphasis on the tens digit when solving multiplication problems, than Italian-speaking children.

## Introduction

Solving multiplication problems is one of the basic arithmetic operations children learn in their school career. The knowledge of these problems is important for other tasks such as division and some addition tasks (i.e. Campbell, 1999; Mauro et al., 2003). However, the multiplication process is error prone, even in highly proficient adults, with a majority of those errors being operand errors (Campbell, 1997; Campbell & Graham, 1985; Domahs et al., 2006, 2007; LeFevre et al., 1996). Operand errors are errors, where false results are related to the same table as one of the operands (e.g., 3 × 6 = 12, 3 × 6 = 24). Previous research has shown that various factors such as problem size (Campbell & Graham, 1985; LeFevre et al., 1996) or numerical closeness of a false to the correct result (Campbell, 1997) have an influence on operand errors.

Operand errors can occur because simple multiplication facts are stored in associative networks of the declarative memory and retrieved from there (e.g. Campbell, 1995; Koshmider & Ascraft, 1991; Verguts & Fias, 2005). Previous research suggests that even children already built those associative networks for multiplication facts (i.e. Cerda et al., 2019; Koshmider & Ashcraft, 1991). These semantic networks lead to neighborhood effects, meaning that when a multiplication problem is given, not only nodes for the correct answer but also neighboring nodes are activated (Verguts & Fias, 2005). The multiplication problem 3 × 6 therefore activates the 8-node as it is the unit digit of the correct answer as well as the 1-node as the decade digit. The result 18 now shares its decade digit with associated neighboring problems, which results in a weaker activation of results such as 12 (3 × 4) and 15 (3 × 5) and might lead to an incorrect answer of the problem. The incorrect retrieval of multiplication problems is also connected to two other important factors, table-relatedness of the erroneous result with the correct one and decade-consistency (Domahs et al., 2006, 2007). To be able to process multi-digit numbers, as in possible results for simple multiplication problems, one has to understand that the Arabic number system is organized in a place-value structure, meaning that every quantity in a number is represented by one symbolic Arabic digit (Chan et al., 2014). The value of one digit depends on its position in the number, e.g. the digit 3 in the hundreds position has a different value than the 3 in the units position. This place-value understanding can be majorly influenced by language (e.g. Bahnmueller et al., 2018). Linguistic factors such as the inversion property of number words (e.g., 37 is spoken siebenunddreißig in German, which translates to seven and thirty; compared to non-inverted languages such as English, where 37 is spoken thirty-seven) moderate number processing and might be responsible for some operand errors associated with solving multiplication problems (Göbel et al., 2014).

Place-value understanding and linguistic influences on it were picked out as central theme in different tasks. Yet, multiplications, although they are highly associated with language (see for the Triple-Code-Model: Dehaene, 1992; Dehaene & Cohen, 1995; for the influence of language on basic multiplication problems: e.g. Chochon, Cohen, Van de Moortele & Dehaene, 1999; Lee & Kang, 2002; but see Amalric & Dehaene, 2016, 2019 for the dissociation of language and mathematical processing), have not received a lot of attention in this category. Previously, Bahnmueller et al. (2020) have focused on the link between linguistic differences in processing place-value information during multiplication tasks in adults.

The studies on operant effects conducted in the context of multiplication fact retrieval (Bahnmueller et al., 2020; Domahs et al., 2006, 2007) show the data for adults. However, little is known about whether the same problems can be found and replicated in children, who have not yet been able to build semantic networks for multiplication as strongly as adults. As of now, effects such as decade-consistency and table-relatedness, as well as associated linguistic effects, cannot be generalized yet in different age groups. We aim to fill this important research gap with the current study by mostly replicating the Bahnmueller and colleagues’ study, in a sample of German and Italian elementary school children.

## Place-value processing and solving multiplication problems

To get a full understanding of how multiplication problems are solved and how effects of neighborhood consistency, decade-consistency, and table-relatedness in multiplication fact retrieval can occur, we first give insight into place-value processing.

When solving arithmetic problems with multi-digit numbers the mental representation of these numbers is in focus (Nuerk & Willmes, 2005). Understanding and activating place-value information is required for many different arithmetic tasks and early place-value understanding even predicts later arithmetic abilities (Moeller et al., 2011). Number magnitude comparisons for example require the knowledge of position and value for each digit of a multi-digit number (Nuerk, Moeller & Willmes, 2015). Other tasks need the ability to make changes to value and/or place. The need for a carry operation in an addition task is an example for place-value computation, since the operation can only be solved correctly when the decade-digit of the unit sum is added to the sum of the decade-digits (e.g. for 27 + 16, 7 + 6 = 13, now the sum of the decade-digits has to be extended, i.e. 2 + 1 + 1 = 4) (Nuerk et al., 2015).

Despite the ongoing discussion on whether multi-digit numbers are processed holistically (each digit pair processed together as one number) or componentially (each digit pair processed separately as a decade- and a unit-digit). In a broad variety of tasks, evidence clearly speaks for a componential processing approach with a decomposed representation for units, tens, hundreds, etc. (e.g., Macizo & Herrera, 2010; Nuerk et al., 2001). Carry operations in addition tasks are a very good example for componential processing of place-value. For these problems (e.g. 18 + 27) it is not sufficient to summate tens and units column by column. Rather, it is necessary to consider the decade-digit of the unit sum (i.e. 8 + 7 = 15) when calculating the sum of the tens (i.e. 1 + 2 + 1 = 4) (e.g. Deschuyteneer et al., 2005; Klein et al., 2010; Nuerk et al., 2015). This clearly reflects the componential processing of multi-digit numbers and indicates that the componential processing of multi-digit numbers is rather the rule that can be generalized over a variety of numerical tasks. Also, the decade-consistency effect found by Domahs et al., (2006, 2007) is an example for componential processing of tens and units in multiplication fact retrieval. In their verification task adult participants were presented a multiplication problem (e.g. 3 × 6). Predominantly table-related errors (3 × 6 = 12) were more likely than table-unrelated errors (3 × 6 = 13). In addition to this table-related effect, Domahs et al. (2007) stated that within the multiplication table other factors are important for the correct solution of the task. The decade of an incorrect result compared to that of the correct result plays a significant role in this context. It was found that 12 is more likely to be falsely chosen as the correct result than 24. Although both options are table-related, 12 falls in the same decade as the correct result 18 and is therefore decade-consistent.

Operand-errors as described by Domahs et al. () are likely to occur, because multiplication facts are found to be stored in associative networks and retrieved from them. When information is needed, for example the result to the multiplication problem 7 × 4, not only the given problem and the correct response are activated, but also neighboring problems (e.g. 7 × 3, 7 × 5, 6 × 4, and 8 × 4) and their results will be represented to some degree (Verguts & Fias, 2005). These associative networks are strongly connected in adults. However, already children, who just learn multiplication tables, solve multiplication problems from direct retrieval and built semantic networks for multiplication facts (Cerda et al., 2019; Koshmider & Ashcraft, 1991). Campbell and Graham (1985) were able to show, that even in early stages of multiplication fact learning (2nd grade), children made similar errors to adult participants. By grade 5 children’s error patterns even mirrored those of adults. They describe learning multiplication skills as associative bonding in a network structure, starting with relatively weaker networks that get stronger with experience (Campbell & Graham, 1985).

## Influences of language on numerical processing

In solving multiplication problems, also language plays an important role and influences how multiplication facts are stored and retrieved. Language effects for arithmetic have already been documented in various areas. Verbal information in general has been shown to influence the way of processing numerical information (Macizo, Herrera, Román & Martin, 2011). Language. However. is not only important in encoding arithmetic problems, but also in nonverbal numerical (symbolic Arabic) tasks, where language-specific differences in number words influence number processing (Bahnmueller et al., 2018; Göbel et al., 2014; Lonnemann & Yan, 2015). This influence extends from symbolic magnitude comparisons (Moeller et al., 2015; Nuerk et al., 2005) to mental addition (Göbel et al., 2014; Van Rinsveld et al., 2015; but also see Brysbaert et al., 1998). With respect to multi-digit number operations the inversion property of number words plays an important role. Inversion includes language properties, which do not follow the Arabic place-value structure for multi-digit numbers. Verbal influences, such as the inversion property, were found in different number processing and arithmetic tasks in children (e.g. Klein et al., 2013; Zuber et al., 2009) and adults (e.g. Lewis et al., 2020; Lonnemann & Yan, 2015). As an example for inversion effects, Nuerk et al. (2005) tested the unit-decade compatibility effect in a German- and English-speaking sample. In the task participants have to decide between a number pair. Pairs are considered unit-decade-compatible when tens and units of a number lead in the same direction (e.g. 42_57: 4 < 5, 2 < 7). A comparison in unit-decade-incompatible pairs leads to contrary response biases (e.g. 47_62: 4 < 6, 7 > 2). Compatible numbers are usually responded to faster and with less errors than incompatible numbers. This effect indicates that the digits of a two-digit number (meaning tens and units) are processed separately. Though the unit-decade compatibility effect was found in English- and German-speaking individuals, it was more pronounced in German-speaking (Nuerk et al., 2005). These findings can be explained by differences in numerical processing due to the differences in the verbal number word systems. The English number word system, but also the number word systems of other non-inverted languages like Italian, are fairly consistent for two-digit numbers (at least ≥ 20). In German, two-digit numbers are inverted with respect to the Arabic digit notation (23 is spoken *dreiundzwanzig*, which translates to *three-and-twenty*). This means that in German the unit-digit of a two-digit number is named first. Thus, it is assumed that the unit-digit also plays a superior role in number processing for German speakers; more than it would in a language without inversion property.

Bahnmueller et al. (2020) first used the link between language, specifically inversion-related effects, and place-value processing. They evaluated the decade-consistency effect and table-relatedness for multiplications in adults with different linguistic backgrounds. A comparison of German- and English-speaking participants revealed that the decade-consistency effect (Domahs et al., 2006, 2007) was more pronounced for English- than German-speaking participants for table-related probes. This indicates that linguistic specifics have an influence on place-value processing, specifically the decade-digit. In English number words the decade-digit is named first, whereas the order of tens and units is inverted in German number words.

## Objectives

Previous research on multiplication fact retrieval has shown that this process can be very error-prone. Most frequently, adults show operand-related errors, with decade-consistency and table-relatedness playing a major role (Domahs et al., 2006, 2007). These errors can be explained by the fact that multiplication facts are stored in semantic networks, in which neighboring information is also activated and thus influences the fact retrieval process (Koshmider & Ascraft, 1991; Verguts & Fias, 2005). Whether operand-related effects are already evident in children, has not yet been investigated.

In adult samples, operand-effects in arithmetic fact retrieval have been studied in more detail, with Bahnmueller et al. (2020) showing that linguistic effects may play a crucial role. In particular, the inversion effect for multi-digit numbers can affect place-value processing and influence the occurrence of operand-related effects. The inversion effect has already been shown in previous research on number processing and arithmetic in adults (e.g. Lewis et al., 2020; Lonnemann & Yan, 2015) as well as children (e.g. Klein et al., 2013; Zuber et al., 2009). It has not yet been investigated, yet, how it affects multiplication fact retrieval in children.

## The present study

While linguistic effects were shown to influence children and adults, it is less clear whether operand-effects in multiplication fact retrieval are the same in different age groups. Previous research is primarily focused on decade-consistency and table-relatedness effects in adult populations.

The current study now aims to address these research gaps and evaluate two main aspects.

We first aim to evaluate, whether place-value effects in multiplication fact retrieval, like the decade-consistency and table-relatedness effects already show in children who just learned multiplication tables. In addition, language specificities will be assessed in this context. This study is intended to replicate the study by Bahnmueller et al. (2020) in a sample of children.

Since adults and children show differences in neural correlates for fact retrieval (Prieto-Corona et al., 2010), we cannot simply transfer ideas from adults to children. Therefore, we believe that our study makes an important impact on multiplication tasks being evaluated in children.

Though, not much is known about operand-related effects regarding multiplication fact retrieval in children, we expect to replicate findings by Domahs et al., (2006, 2007), who claim that items that include decade-consistent distractors will be solved slower than multiplication problems with decade-inconsistent lures. Table-related lures should be harder to reject than lures that are not related to the table of one of the operands. Previously, operand-effects were linked to place-value processing and associative networks for multiplication facts in adults (e.g. Campbell, 1995). But also children are believed to store multiplication facts in semantic networks and retrieve them from there (Campbell & Graham, 1985), even if they might not be as strong as in adults. Therefore, operand-effects should show in children, even if they might be weaker.

Bahnmueller et al. (2020) further contributed to the influences of language on place-value processing by considering the decade-consistency effect. They found that the effect was more pronounced for English-speaking adults than German-speaking. We assume that the language-specific differences that were found in adults will already show in children, as inversion-effects have been shown in both children and adults in previous research (i.e.Klein et al., 2013; Lonnemann & Yan, 2015). For comparability with the results by Bahnmueller et al. (2020) we tested German- and Italian-speaking children. In Italian, just like in English, the order of tens and units in number words follows the order of the Arabic notation, whereas in German number words the order is inverted, which results in different foci concerning place-value structure in the different languages. Consequently, we follow the hypothesis that the processing of decade-digits might be more enhanced for Italian-speaking children because in Italian number words the decade-digit is named first whereas in German number words the unit-digit is named first.

Although we replicate Bahnmueller et al.'s (2020) study in children and mostly expect parallels, we also see the possibility of differences in how effects will show. Following Domahs et al. (2006), Bahnmueller et al., were able to show effects in error rates, but not clearly in RTs.

This inconsistent picture might also be evident in our sample with children. However, following previous research, we would expect this to be contrary to Bahnmueller et al. (2020). Koshmider and Ashcraft (1991) found in their study that reaction time effects were clearer for younger children (third grade) compared to older children (seventh grade). Operand-related errors on the other hand seem to increase with age (Butterworth et al., 2003) and therefore might show more clearly in error rates.

## Methods

### Participants

A total of 58 primary school children participated in the study. Nine participants had to be excluded due to missing data from the computer task. Three additional children were eliminated because their overall error rate exceeded 33% (Bahnmueller et al., 2020). The remaining participants are divided into 22 participants in the German language group and 24 participants in the Italian language group. All participating children were monolingual native speakers in their respective language. The 22 German-speaking children were on average 8; 95 years old [SD = 0.41, 14 female]. Italian-speaking children were 8; 74 years old [SD = 0.33, 9 female]. Irrespective of age, all participating children in our study were in third grade, end of first term, at the time of data collection. German speaking children were recruited from schools in Innsbruck, Austria. Children with Italian as their native language were tested in schools in Trentino-South Tyrol. All participants were native speaker of their respective language. Importantly, the mathematics curriculum is very similar in Austria and Italy (cf. mathematics curricula from Bundesministerium für Bildung, Wissenschaft und Forschung and Ministero dell'Istruzione, Ministero dell'Università e della Ricerca). Therefore, all children were first introduced to multiplications at the end of second grade/beginning of third grade and had already learned all basic multiplication tables (all tables from 1 to 10).

### Task, stimuli and design

Participating children were asked to solve multiplication problems in a single-digit times single-digit format with two-digit results. For comparison purposes the carefully balanced stimulus set of Domahs et al. (2007) was used. However, the presentation of stimuli was different. Domahs et al. (2007) (as well as Bahnueller et al., 2018) made use of a verification task, in which adult participants decided whether the presented result was correct or not. In our elementary school students, we presented all multiplication problems with two results and children had to choose the correct result from two presented options (with one correct and one incorrect). We decided to present items in this form because children in third grade of elementary school are usually not yet proficient with multiplication problems and relatively prone to distractions. We therefore intended to lower the number of irregular errors made due to inexpertness with multiplication tables. We also decided on this format for economic reasons, as we wanted to keep the experiment fairly short so the children would stay concentrated and motivated.

Regarding the use of lures we stuck to the manipulation suggested by Domahs et al. (2007), who utilized a 2 × 2 design with the factors relatedness (table-related vs. not table-related) and decade-consistency (decade-consistent vs. decade-inconsistent). On the basis of eighteen different multiplication problems, 72 different items were created with the four different lure types (related and consistent, related but inconsistent, unrelated but consistent, unrelated and inconsistent). As an example, for the multiplication problem 3 × 6, the related and consistent lure was 12, the related but inconsistent lure was 24, the unrelated but consistent lure was 13 and the unrelated and inconsistent lure was 23. Accordingly, all eighteen multiplication problems were presented four times with the correct solution in combination with either of the lure conditions. For a complete list of items and examples for lures see Domahs et al. (2007).

During the experiment responses and reaction times in ms were recorded for all items.

### Procedure

The experiment was held in single settings in separate rooms directly at children’s schools. Both language groups were given the same experiment with the same procedure.

**Fig. 1:**
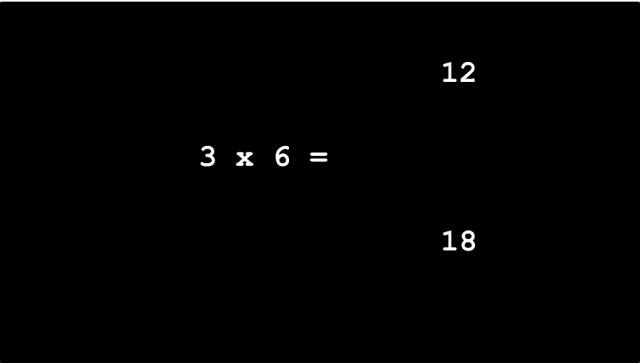
Exemplary illustration of the experimental task

After obtaining written consent by parents or legal guardians and the children themselves, they were instructed to decide as quickly and accurately as possible between two solution probes for a presented multiplication problem. In case the probe presented on top was correct children should press the “z” key on a standard QWERTZ keyboard. To choose the bottom probe as correct, the “n” key had to be pressed. Both keys were clearly marked for the children. The experiment was presented on a 17″ laptop screen driven at a resolution of 1600 × 900 pixels. Stimuli were presented in white against a black background in Courier New (bold, font size: 48). The two operands were presented simultaneously and were separated by an “x” for the multiplication sign. Two possible probes were given among each other after the equal sign (see Fig. 1).

A fixation cross preceded each trial and was presented for 500 ms in the middle of the screen. The multiplication problem was displayed until a decision was made by the participant (keypress “z” or “n”). Trials were separated by an interval of 500 ms.

The experiment with all 72 items started after five practice trials. Practice trials were also the same as in Domahs et al. (2007). Trial order was randomized individually for each participant. In total the experiment lasted approximately 15 min.

### Data analysis

A 2 × 2x2 ANOVA (including the within-subject factors decade-consistency, relatedness and the between-subjects factor language group) was conducted for reaction times. Data was analyzed with the statistical software IBM SPSS Statistics 25. The *p*-value was set at *p* ≤ 0.05.

Unlike Domahs et al. (2006) and Bahnmueller et al. (2018), we have decided against error rates in the reporting of results. Before data was analyzed, we checked for outliers in RTs and error rates cleaned. Error rates showed ceiling effects, as an average of 67.39 out of 72 items were solved correctly. This corresponds to an average accuracy rate of 93.6%. Therefore, we will focus on findings regarding reaction times as a means of presenting important results.

Problems were only entered into the analyses when the items were solved correctly. Additionally, reaction times were excluded separately for every participant when they deviated from the item’s overall mean response time by more than ± 3SD.

## Results

The mixed-model ANOVA (see Table 1. for mean values of all stimuli) showed no significant difference between language groups [*F*(1,44) = 2.08, *p* = 0.156, *η*_*p*_^2^ = 0.045; German: *M* = 4946 ms, SD = 344 ms; Italian: *M* = 4259 ms, *SD* = 330 ms]. A main effect for decade-consistency was found [*F*(1,44) = 5.42, *p* = 0.025, *η*_*p*_^2^ = 0.110]. Accordingly, responses were faster to items with decade-inconsistent lures [*M* = 4472 ms, *SD* = 242 ms] than to items with decade-consistent lures [*M* = 4733 ms, *SD* = 248 ms]. The interaction between decade-consistency and language group did not yield significant results [*F*(1,44) = 0.02, *p* = 0.884, *η*_*p*_^2^ = 0.000].

**Table 1:** Mean values *M* and standard deviations (*SD*) for stimuli categories used in the ANOVA

	German	Italian
Decade consistent		
Table related	5088 (398)	4827 (382)
Table unrelated	5049 (354)	3969 (339)
Decade inconsistent		
Table related	5029 (411)	4330 (393)
Table unrelated	4618 (322)	3909 (308)

Furthermore, we found a reliable main effect for the factor relatedness [*F*(1,44) = 10.54, *p* = 0.002, *η*_*p*_^2^ = 0.193], indicating that items with table-related lures were solved significantly slower [*M* = 4819 ms, *SD* = 267 ms] than items with table-unrelated lures [*M* = 4472 ms, *SD* = 226 ms].

However, no significant interaction for relatedness and language group was found [*F*(1,44) = 2.42, *p* = 0.127, *η*_*p*_^2^ = 0.052]. Also, the interaction for decade-consistency and relatedness did not yield significant results [*F*(1,44) = 0.03, *p* = 0.867, *η*_*p*_^2^ = 0.001].

**Fig. 2:**
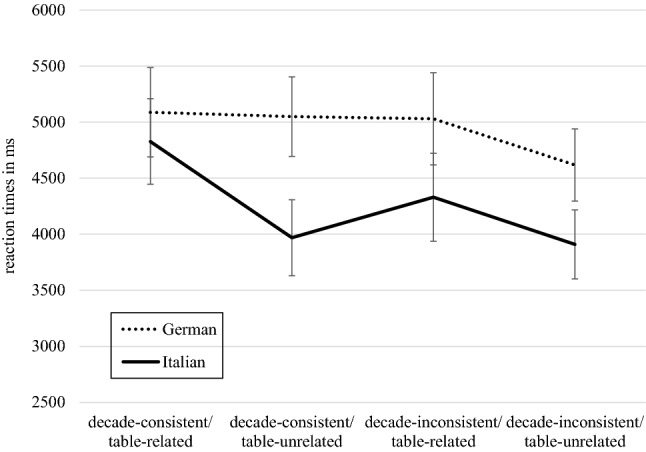
Display of response times in ms in German and Italian children for all item categories

We found a significant three-way interaction for relatedness, decade-consistency and language group for reaction times [*F*(1,44) = 4.45, *p* = 0.041, *η*_*p*_^2^ = 0.092] (see Fig. 2.). Breaking down this three-way interaction, into the two two-way interactions of table-relatedness and language group for decade-consistent and decade-inconsistent problems, it is revealed that the two-way interaction of relatedness and language group was significant for decade-consistent [*F*(1,44) = 6.13, *p* = 0.017, *η*_*p*_^2^ = 0.122] but not for decade-inconsistent items [*F*(1,44) = 0.00, *p* = 0.978, *η*_*p*_^2^ = 0.000]. For decade-consistent problems the effect of table-relatedness was significant for the Italian language group [*t*(23) = 3.15, *p* = 0.004] but not for the German language group [*t*(21) = 0.22, *p* = 0.827]. For German-speaking children mean reaction times reveal, that items from all categories were solved relatively the same [*M*_decade-consistent/table-related_ = 5088 ms, *SD*_decade-consistent/table-related_ = 1690 ms; *M*_decade-consistent/table-unrelated_ = 5049 ms, *SD*_decade-consistent/table-unrelated_ = 1730 ms; *M*_decade-inconsistent/table-related_ = 5029 ms, *SD*_decade-inconsistent/table-related_ = 1766 ms], except for decade-inconsistent and at the same time table-unrelated lures, that were solved the quickest [*M* = 4618 ms, *SD* = 1360 ms].

The mentioned two-way interaction indicates that differences in reaction times between table-related and –unrelated items were only significant for Italian children (see Fig. 2).

## Discussion

The present study aimed to replicate Bahnmueller et al.’s (2020) findings on operand-related effects in multiplication fact retrieval and thee influence of language specificities, in children. In a task of choice German- and Italian-speaking elementary school children had to decide on the right solution for a given multiplication problem. The focus was on the evaluation of decade-consistency and table-relatedness.

As we expected, the results in our sample appeared partially comparable to the findings of Bahnmueller et al. (2020). However, there were clear ceiling effects for error rates, so that we could only draw comparisons on the basis of reaction times, which are not explicitly comparable to the results from Bahnmueller et al. Yet, according to Koshmider and Ashcraft (1991), reaction times effects are more evident for younger children than for older ones. For this reason, we allow ourselves to draw comparisons between Bahnmueller et al. (2020), Domahs et al., (2006, 2007), and our current sample. Overall, a comparable pattern was found between adults and children in terms of operand-effects and linguistic influences. Yet, this is reflected in error rates for adults and RTs for children.

Our findings in a sample of elementary school children were generally comparable to Domahs et al., (2006, 2007) regarding the decade-consistency and table-relatedness effects. Additionally, we were able to show clear linguistic influences on place-value processing (cf. Bahnmueller et al., 2020). Differences between German and Italian children suggest the automatic activation of place-value information when multi-digit numbers have to be processed and additionally strong linguistic influences caused by a difference in number word structures.

Overall, effects of decade-consistency and table-relatedness in fact retrieval were found in children, though the effects were weaker than in adults (i.e. Bahnmueller et al., 2020). We suppose that these effects are linked to automatic place-value activation during arithmetic fact retrieval (e.g., Bahnmueller et al., 2020) which happens in children and adults. As Campbell and Graham (1985) suggested children already store multiplication facts in semantic networks, even if they are not as strong as in adults. Therefore, children seem to show weaker operand-effects, that will grow stronger till adulthood, where higher proficiency with place-value processing and stronger associative multiplication networks lead to stronger effects in operand-related questions.

Linguistic specificities and therefore differences between the language groups became noticeable when looking at the factor of table-relatedness in decade-consistent probes. While items with decade-inconsistent lures were responded to with relatively equal RTs in both language groups, regardless of whether they were table-related or not. A rather different pattern was shown for items with decade-consistent lures. Whereas German children seemed to be less influenced by the table-relatedness, Italian children showed significant differences in responses to either table-related or table-unrelated lures, when items were also decade-consistent with the correct answer. For German children we saw that all item categories, except decade-inconsistent table-unrelated lures, were solved relatively the same looking at reaction times. Hence, they do not seem to be highly affected by decade-consistency or table-relatedness in general. The exception of decade-inconsistent table-unrelated lures being detected the quickest can be explained logically. Lures that show a different decade than the correct solution and are not related to the table of one of the operands, are the first option that can be eliminated and therefore are excluded first. This suggestion is in line with findings for example by Verguts and Fias (2005), who claim that multiplication facts are organized in a semantic network structure. So, whenever a multiplication problem is given, usually the correct result and a number of neighboring nodes in the network are activated. A solution that is neither decade-consistent nor table-related with the correct result therefore should not be directly associated with the correct result and easily be rejected. We were not surprised, that for decade-consistent lures no effect of table-relatedness could be found in German-speaking children. The inversion of number words (in German 23 is spoken dreiundzwanzig which literally translates to three and twenty) highly affects number processing (e.g., Göbel et al., 2014). In our study, German-speaking children might give less importance to the tens digit when solving multiplication problems but look at the unit digit first, as they would do while speaking out loud. Therefore, they are not affected by changing decades and consequently are less influenced by decade-consistent lures, table-related or not. The fact that table-relatedness in items with decade-consistent lures was only found for Italian-speaking children corroborates our hypothesis that decade-digit processing while solving multiplication problems might be more enhanced for the Italian cohort. As the Italian language does not have inverted number words for two-digit numbers as in German, numbers seem to be processed differently. We suppose that Italian-speaking children put more emphasis on the tens digit and therefore the table-relatedness effect is more pronounced for these children, when the given lure has the same tens digit as the correct result. When Italian-speaking are given such a problem, they now cannot simply compare the tens digits for solving the problem but have to pay attention to the multiplication table. If the tens digit of the wrong result matches the right one, you have to pay more attention to other characteristics. Thus, the decision between table-related and -unrelated is associated with longer reaction times. If the tens digit of the lure is different, then no time needs to be spent on differentiation, because the result can be rejected immediately. This explains why the table-relatedness effect was not found for decade inconsistent items. The found inversion-related influences on place-value processing in solving multiplication problems, were in line with previous research with adults (Bahnmueller et al., 2020) and indicate, that multiplication fact retrieval is already vulnerable to linguistic specificities in children.

However, our study also shows some limitations and provides further ideas for the research on language differences in children's place-value understanding. Although we used the same set of items as Domahs et al. (2006) and Bahnmueller et al. (2020), we opted for a different presentation design in our sample due to economic reasons. Either the change of presentation format or our much younger sample might have let to ceiling effects in error rates and let us only draw comparisons based on reaction times, which are not explicitly comparable to Bahnmueller et al. In further research, a direct replication of the study design of the above studies should definitely be considered. This could be aimed at with slightly older children. Moreover, in the current study we found comparable results with Domahs et al., and Bahnmueller et al., especially in the children's reaction times. Yet, the effects were much weaker than in adults and could be evaluated again with a bigger sample size.

### Conclusion and future perspectives

In conclusion, the findings of the present study confirm operand-effects, showing in decade-consistency and table-relatedness, in multiplication fact retrieval in children. Even though effects were not as strong as in adult participants (i.e. Bahnmueller et al., 2020), they showed significant relevance and should be considered in future research. As a practical implication, the topic of operand-processing and associated effects in children, should be more considered in educational settings. Teachers should be made aware of this topic, and accordingly be able to react to problems by modifying tasks etc. In addition, inversion-related influences on place-value processing in arithmetic fact retrieval in children were found. It was clearly shown that German-speaking children are much less influenced by effects related to the place-value structure of multi-digit numbers. Italian-speaking children, on the other hand, are very much influenced by lures, which aim at the tens digit of numbers. Children with different language backgrounds therefore have different problems, which cannot simply be transferred between the languages. This was already shown in previous inversion studies and is now specifically manifested for effects regarding arithmetic fact retrieval. We could successfully show that interactions between the processing of place-value information during arithmetic fact retrieval and language-based effects already occur in children. These results are particularly relevant in terms of understanding linguistic differences and the associated specifics and their consideration in didactics. This knowledge can function as a basis for education and support programs in bilingual children, who are influenced by different language specificities in fact retrieval. More knowledge in this direction would help to understand difficulties that arise for children who are confronted with two languages in their learning environment, and would be an idea for future research. It would also be highly interesting to take an even closer look at the topic of multiplication fact retrieval and to replicate our findings with the help of eye-tracking, to further confirm our conclusions.
